# Lithium-Ion Conduction in Liquid-Crystalline Columnar Pd(II) Nanoassemblies

**DOI:** 10.1021/acsami.5c00209

**Published:** 2025-06-12

**Authors:** Cristián Cuerva, Irene Caro-Campos, Mercedes Cano, Enrique Rodríguez-Castellón, Alois Kuhn, Flaviano García-Alvarado, Rainer Schmidt

**Affiliations:** † Departamento de Química Inorgánica, Facultad de Ciencias Químicas, 16734 Universidad Complutense de Madrid, Ciudad Universitaria, E-28040 Madrid, Spain; ‡ Departamento de Química Inorgánica, Facultad de Ciencias, Instituto Interuniversitario de Investigación en Biorrefinerías I3B, Universidad de Málaga, 29071 Málaga, Spain; § Departamento de Química y Bioquímica, Facultad de Farmacia, Universidad San Pablo-CEU, CEU Universities, Urbanización Montepríncipe, Boadilla del Monte, 28668 Madrid, Spain; ∥ GFMC. Departamento de Física de Materiales, Facultad de Ciencias Físicas, 16734 Universidad Complutense de Madrid, Ciudad Universitaria, E-28040 Madrid, Spain

**Keywords:** metallomesogens, liquid crystals, Li-ion conduction, nanoassemblies, columnar mesophase

## Abstract

Liquid crystalline electrolytes are emerging as a promising class of functional materials for energy storage applications. They offer the ability to operate under anhydrous conditions without the presence of acids or flammable solvents, allowing high operating temperatures. Herein, the liquid crystalline phase of a bispyrazolate Pd­(II) metallomesogen is used as a platform for Li-ion conduction, taking advantage of the existence of nanochannels in the hexagonal columnar mesophase. Li-doped liquid crystal composites have been prepared with different lithium content, and their mesomorphic properties and ionic conductivities were studied. It was found that the intercalation of lithium ions between molecules does not hinder the formation of the mesophase but rather extends the temperature range in which it is stable due to the existence of ion–dipole interactions between the lithium ions and the uncoordinated N-pyrazolic atoms, leading to lower melting and higher clearing temperatures. High Li-ion conductivity was found in the solid and liquid crystalline phases by complex impedance spectroscopy. The optimally doped composite with an 8:2 (metallomesogen:LiTFSI) molar ratio reaches conductivity values as high as 1.89 × 10^–4^ Ω^–1^ cm^–1^. The work presented is expected to pave the way for a promising class of liquid crystalline Li-ion electrolytes based on metallomesogens.

## Graphical abstract

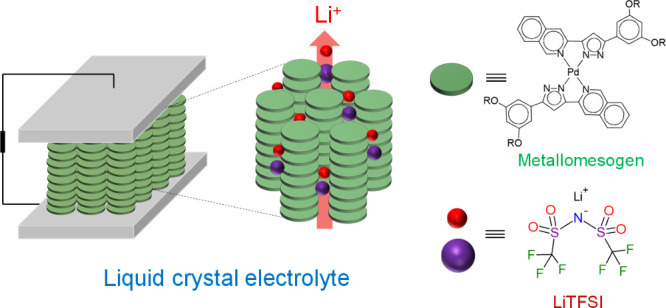

## Introduction

Nanostructured liquid crystals are emerging as a new type of ionic conductors for potential energy applications.^1–3^ The combination of long-range directional order and fluidity makes them excellent candidates for the transport of ions.^4^ In this type of materials, the conduction pathways are determined by the supramolecular organization in the liquid-crystalline mesophase.^5,6^ Thus, columnar mesophases often act as one-dimensional conductors, whereas smectic and bicontinuous cubic mesophases are usually used for two- or three-dimensional ionic transport.^7–9^

Ionic liquid crystals have been widely studied as quasi-solid-state electrolytes.^10–12^ Nanosegregation of the aromatic moieties containing the alkyl chains and the counterions leads to aligned layers or nanochannels for high ionic conductivity.^13–15^ In neutral compounds that do not contain charged species, the liquid-crystalline phase can be used as a soft platform for the transport of charged species via ion-doping. The fabrication of these composites offers the possibility to modulate the doping concentration, which in turn allows control of the ion content in the nanostructures and, concomitantly, the ionic conductivity.^16–18^ The conductivity of Li^+^ ions in these metallomesogens facilitates their use as electrolytes in Li-ion batteries.^18^

Liquid crystals also offer the advantage of high operational temperatures, because humid conditions are not required for the ion transport to occur.^19^ In this context, metallomesogens are an excellent option to form ion conductive mesophases with wide stability ranges.^20^ In previous studies, proton conduction at high temperatures under anhydrous conditions has been reported in columnar mesophases of bispyrazolate Pd­(II) and Pt­(II) metallomesogens.^21–24^ The conductivity values obtained in these compounds were rather moderate, of the order of 10^–6^ to 10^–9^ Ω^–1^ cm^–1^, because proton conduction was found to be associated with a C–H···N proton transfer that requires high activation energies.^23^ Nonetheless, these metallomesogens form unusually stable Col_h_ mesophases that are of great interest to serve as a general platform for ionic conduction.

Furthermore, in a complementary study it was demonstrated that the introduction of asymmetry in the length of the terminal alkyl chains causes a decrease of the melting temperatures, where compounds with intermediate chain lengths exhibit mesophases with wider stability ranges.^25^ Likewise, the functionalization of the pyrazole ligand with an isoquinoline group had been described as a synthetic strategy that allows increasing the clearing temperatures in these species.^24^ Both these beneficial structural properties, asymmetry in the terminal alkyl chains and the isoquinoline functionalization of the pyrazole ligand, were recently combined in a promising family of novel asymmetrical bis­(isoquinolinylpyrazolate) Pd­(II) metallomesogens, [Pd­(pz^R(12,12)iq^)­(pz^R(n,n)iq^)].^22^ Palladium was selected over other metal centers due to its d^8^ configuration, which would allow reaching the ideal nondistorted square-planar coordination environment required to obtain disc-like molecules with high ability to be self-assembled in columnar mesophases, e.g., via intermolecular Pd···Pd and π···π interactions. Due to the excellent structural stability of these Col_h_ mesophases, which open ordered and continuous nanochannels over a wide temperature range near room temperature, as a next step, Li-ion doping was explored by the incorporation of lithium bis­(trifluoromethane)­sulfonimide (LiTFSI) salt with the aim to induce high one-dimensional Li-ion conductivity. For the current work, the asymmetrical compound shown in Figure 1a (compound **1**) bearing 12 and 14 carbon atoms in the alkyl chains was selected from several candidates of the above-mentioned family [Pd­(pz^R(12,12)iq^)­(pz^R(n,n)iq^)]. This selection of compound **1** was motivated by its superior liquid crystalline behavior in terms of the Col_h_ mesophase reaching down to near room temperature and being highly stable up to 371 °C.^22^ In the following, the synthesis, phase behavior, and Li-ion conductivity of such novel Li-doped metallomesogens are reported in detail.

**Figure 1:**
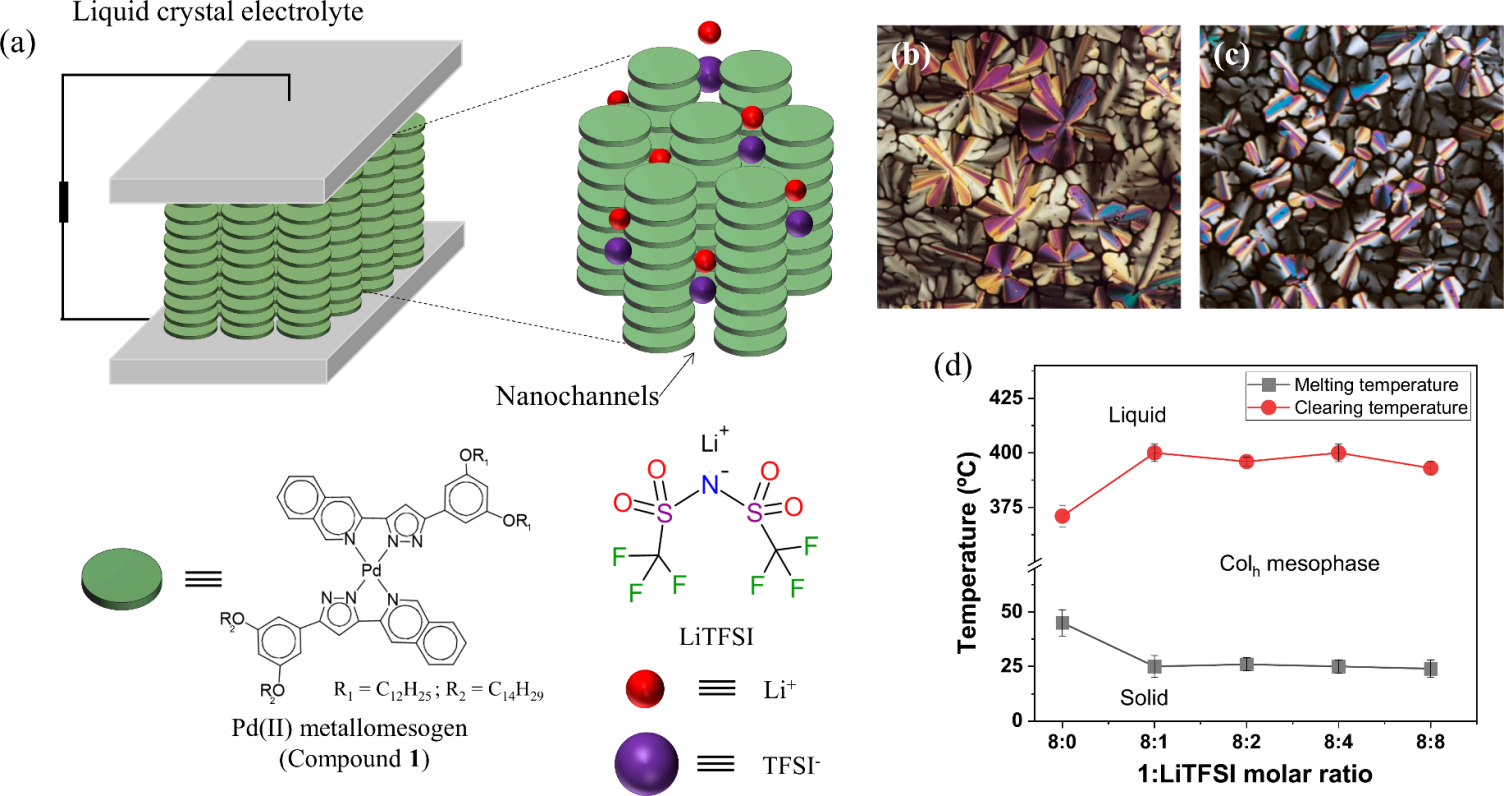
(a) Schematic drawing showing the assembly of the Li-doped liquid crystal electrolytes using a Pd­(II) metallomesogen (compound **1**) as a platform and lithium bis­(trifluoro-methanesulfonyl)­imide (LiTFSI) dopant. (b,c) Mesophase textures observed by POM for the composites prepared with molar ratios **1**:LiTFSI of (b) 8:8 at 90 °C and (c) 8:4 at 110 °C. The images were taken during the cooling cycle using crossed polarizers. (d) Melting and clearing average temperatures determined by POM as a function of the LiTFSI content ± standard deviation of three different measurements.

## Results and Discussion

Li-doped liquid-crystalline composites were produced by slow evaporation of tetrahydrofuran (THF) solutions containing compound **1** and LiTFSI in different molar ratios of **1**:LiTFSI of 8:1 to 8:8. Polarized light optical microscopy (POM) was carried out to reveal the formation of dendritic and pseudofocal conic textures (Figure 1b,c), which indicates that the presence of LiTFSI does not prevent the formation of the Col_h_ mesophase but in fact extends its temperature range. All composites exhibit a mesophase at room temperature, whereas clearing occurs at higher temperatures ranging between 396 and 400 °C as a function of the LiTFSI content (Figure 1d). This decrease in melting temperatures and increase in clearing temperatures with respect to those of compound **1** (45 and 371 °C, respectively) indicates an improvement of the thermodynamic stability and the extension of the temperature range of the mesophase due to the presence of LiTFSI.^22^ Regardless of the molar ratio **1**:LiTFSI, the temperature for the Col_h_-to-isotropic phase transition increases with Li doping. The observed thermodynamic stabilization of the mesophase may most likely be associated with the formation of ion–dipole interactions between the lithium ions and the pyrazolate nitrogen atoms of the ligands, strengthening the columnar assembly of the molecules.^18^

Small-angle powder X-ray diffraction (SAXRD) was performed on the self-assembled liquid crystalline mesophases. Figure 2a shows the XRD pattern of the Col_h_ mesophase at 150 °C for the composite with a 8:2 (**1**:LiTFSI) molar ratio. It displays a series of four sharp peaks that can be indexed as the (100), (110), (200), and (210) reflections of the Col_h_ lattice (*d* = 28.4 Å) (Table 1).^26,27^ Furthermore, two broad peaks appear in the middle-angle region at (i) around 17° (*d* = 5.3 Å) as a result of the molten state of the alkyl chains, and (ii) at around 25° (*d* = 3.4 Å) due to the intracolumnar distance that separates two disc-like molecules from the same column.^28^ Based on these results, it can be concluded that the columnar assembly in the mesophase is most likely driven by intermolecular Pd···Pd and π···π interactions.

**Figure 2:**
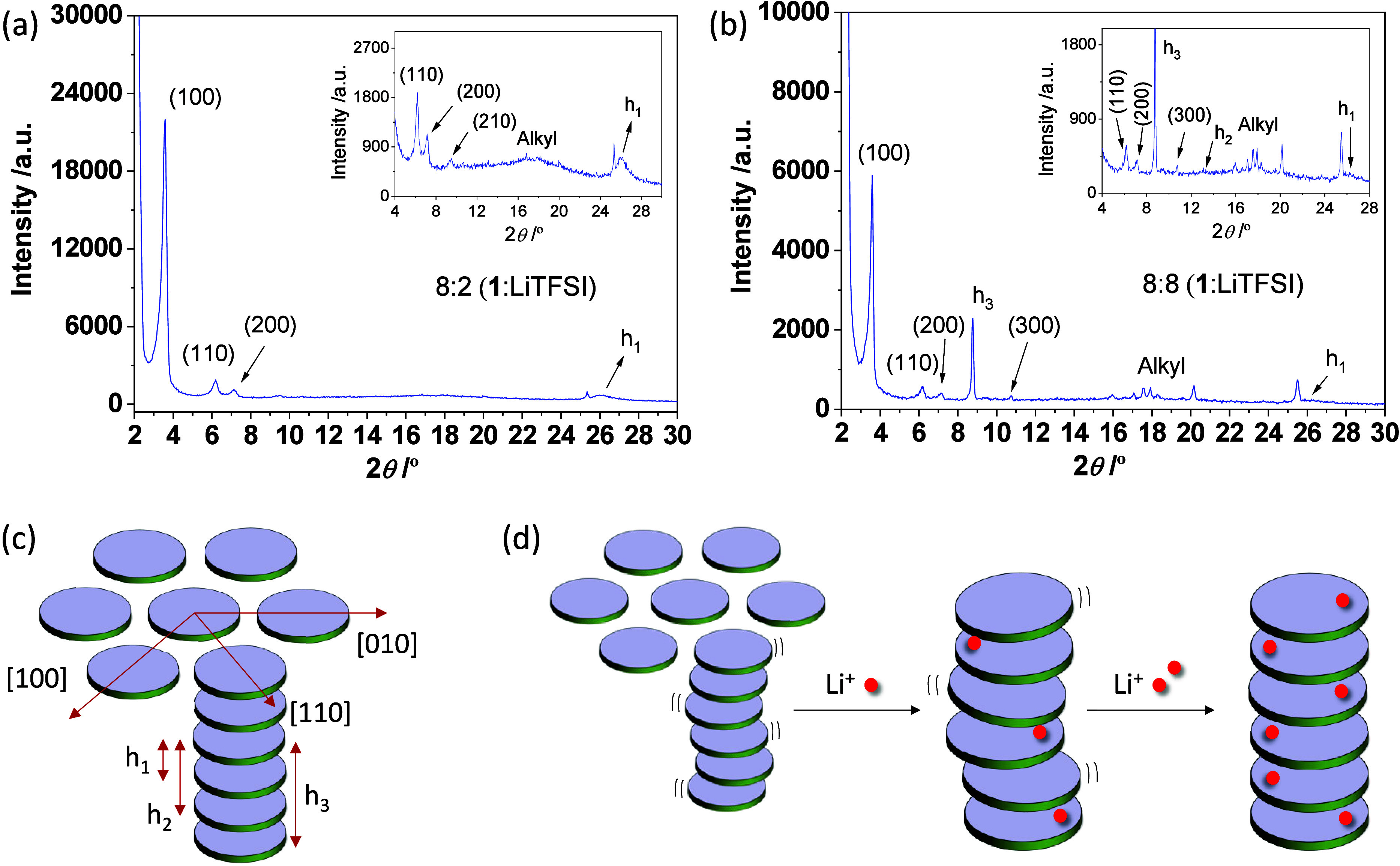
(a,b) XRD patterns from the Col_h_ mesophase in **1**:LiTFSI doped with molar ratios of (a) 8:2 and (b) 8:8 (*T* = 150 °C). The glitch at 25.6 ° is an artifact of the instrument (alumina from the sample holder). (c) Indexation for a hexagonal lattice. (d) Schematic drawing showing the increase of supramolecular ordering in the Col_h_ mesophase in compound **1** after doping with LiTFSI.

**Table 1:** XRD Analysis of the Col_h_ Mesophase at 150 °C

**1**:LiTFSI molar ratio	*d*-spacing (Å)	(*hkl*)^a^	lattice constant^c^
8:0	24.1, 14.1, 12.2, 5.2, 3.4	100, 110, 200, alkyl^b^, h_1_ ^d^	*a* = 28.1 Å
8:2	24.6, 14.2, 12.3, 9.3, 5.3, 3.4	100, 110, 200, 210, alkyl^b^, h_1_	*a* = 28.4 Å
8:8	24.6, 14.3, 12.3, 10.1, 8.2, 6.7, 5.2, 3.4	100, 110, 200, h_3_ ^d^, 300, h_2_ ^d^ alkyl^b^, h_1_ ^d^	*a* = 28.4 Å

a (*hkl*) are the Miller indices of the respective reflections.

b Broad halo associated with the liquid-like order of the molten alkyl chains.

c Lattice constant *a* = 2­[∑*d*_*hk*_√(*h* ^2^ + *k* ^2^ + *hk*)]/√3*N*_*hk*_, where *N*_*hk*_ is the number of *hk*0 reflections.

d h_1_, h_2_, and h_3_ correspond to the intracolumnar distances (00l) between two, three, and four disc-like molecules.

A similar XRD pattern as that described above was reported previously in undoped Pd­(II) metallomesogens,^22^ which suggests that a low **1**:LiTFSI molar ratio such as 8:2 associated with low Li-ion content does not cause remarkable alterations in the supramolecular ordering of the mesophase. However, by increasing the content of lithium, new diffraction peaks appear in the low- and middle-angle regions, as demonstrated in Figure 2b when the **1**:LiTFSI molar ratio is 8:8. Among them, the most noticeable peaks are those at 8.8° (*d* = 10.1 Å), 10.7° (*d* = 8.2 Å), and 13.2° (*d* = 6.7 Å), which correspond to the intracolumnar distances h_3_ and h_2_ as well as the (300) reflection, respectively (Figure 2c, Table 1). Also note the presence of new reflections that appear inside the broad halo of the molten alkyl chains, attributed to *d*-spacings between the molten alkyl chains of neighboring molecules. All of these features are a clear indication of the increase of the long-range order in the Col_h_ mesophase with LiTFSI doping along the column axis, whereas the lattice constant seems hardly affected at all. To confirm that the new reflections correspond to an increase in the long-range order and not to the presence of an excess of pure LiTFSI crystals from the increase in LiTFSI content, the diffraction peaks of LiTFSI have been included in the diffractogram of **1**:LiTFSI (8:8) for comparison (Figure S1).

Most likely, the establishment of the ion–dipole interactions between the lithium ions and the pyrazolate nitrogen atoms of the ligands restrains the axial fluctuations of the molecules and, concomitantly, increases the supramolecular ordering of the mesophase (Figure 2d). These results are consistent with the observed thermodynamic stabilization of the Col_h_ mesophase of compound **1** by Li-ion doping, resulting in an increase in the clearing temperature.

^7^Li MAS NMR measurements were conducted in the solid state to confirm the existence of interactions between compound **1** and the lithium ions. As noted in Figure 3a, the ^7^Li chemical shift of LiTFSI (δ = −1.25 ppm) is strongly downfield shifted in the presence of **1** (δ = −0.33 ppm), which is clear evidence that lithium ions interact with the molecules of the metallomesogen in the composite.

**Figure 3:**
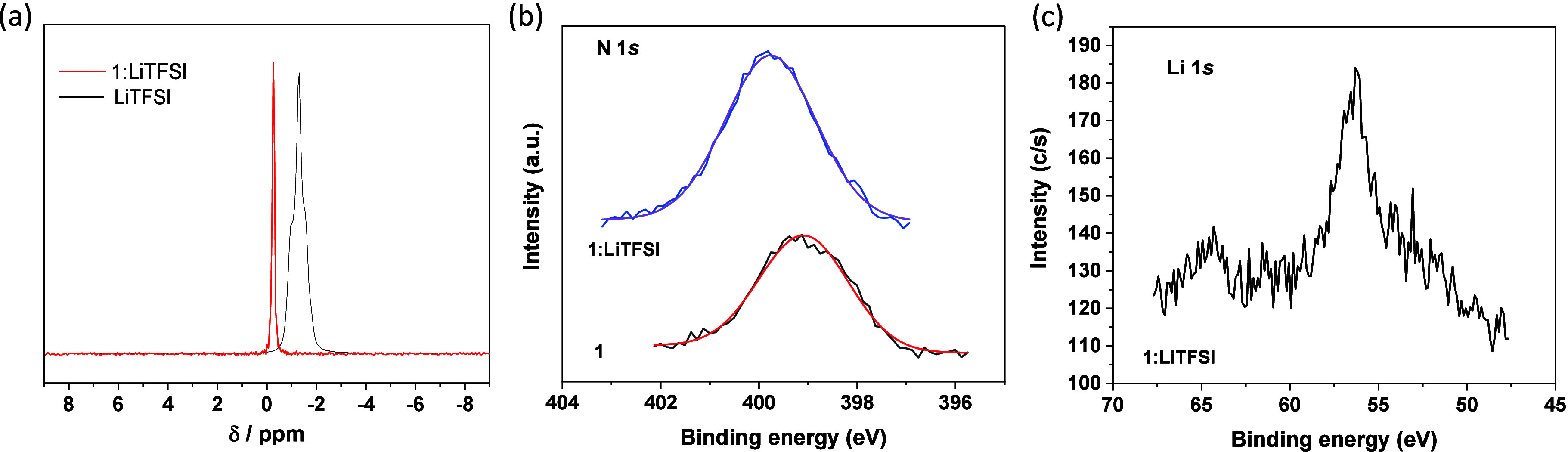
(a) ^7^Li MAS NMR spectra for **1** and **1**:LiTFSI with a 8:8 molar ratio. (b) High resolution N 1s core level spectra for **1** and **1**:LiTFSI (8:8). (c) High resolution Li 1s spectrum for **1**:LiTFSI (8:8).

The surface of samples **1** and **1**:LiTFSI (8:8) was studied by X-ray photoelectron spectroscopy (XPS). The high resolution N 1s core level spectra of samples **1** and **1**:LiTFSI and the high resolution Li 1s spectrum of **1**:LiTFSI are depicted in Figure 3b and 3c, respectively. The N 1s signal of sample **1** is centered at 399.1 eV, while that of sample **1**:LiTFSI appears shifted to 399.8 eV (Figure 3b). This observed shift is due to the interaction of the pyrazolate nitrogen atom with Li^+^, where the negative charge density of the nitrogen atom is shifted toward Li^+^. In addition, the Li 1s signal of sample **1**:LiTFSI appears at 56.0 eV (Figure 3c). This binding energy value is lower than that of LiTFSI (56.7 eV),^29^ where the observed shift is due to the shift of the electron density from the pyrazolate nitrogen atom to Li^+^. Therefore, ^7^Li MAS NMR and XPS measurements confirm the interaction between the lithium ions and the molecules of the Pd­(II) metallomesogen.

The thermal stability of **1**, LiTFSI, and the selected composite **1**:LiTFSI (8:8) was analyzed by differential scanning calorimetry (DSC) and thermogravimetric analysis (TGA). The DSC curve of **1** reported previously displays the endothermic peak associated with the solid-mesophase transition at 45 °C (Δ*H* = 26.8 kJ·mol^–1^).^22^ In the case of LiTFSI salt, three endothermic peaks are observed at onset temperatures of 42 °C (Δ*H* = 2.0 kJ·mol^–1^), 149 °C (Δ*H* = 6.5 kJ·mol^–1^), and 200 °C (Δ*H* = 1.2 kJ·mol^–1^) (Figure S2a). The first peak is associated with the dehydration process of the salt, the second one corresponds to the phase transition between the *transoid* and *cisoid* conformers of TFSI^–^, and the third peak is a result of the melting process.^30–32^ Note that no exothermic peaks are observed upon heating. This is a clear indication that both components of the composites are stable in the operational temperature range of 40–290 °C. In fact, the onset decomposition temperature of each component, relative to 5% weight loss of the initial mass, was established from TGA studies at 307 and 386 °C for **1** and LiTFSI, respectively (Figure S3). Thus, it is unlikely that **1**:LiTFSI composites could decompose at temperatures below 300 °C. As shown in Figure S2b, no signs of sample degradation are observed in the DSC trace of the composite, but in fact decomposition begins to occur at 306 °C as for **1**:LiTFSI (8:8) (Figure S3). It is also noteworthy that the endothermic peak attributed to the characteristic conformational transformation of TFSI^–^ appears at a very similar temperature (143 °C) as in the DSC thermogram of the LiTFSI salt. However, the temperature at which LiTFSI melts in the composite increases to 255 °C. This feature is associated with the ion–dipole interactions between the lithium ions and the pyrazolate nitrogen atoms of the coordinated ligands of **1**.

The charge transport and dielectric properties of all Li-doped liquid crystals were investigated by impedance spectroscopy in a temperature range of 160–560 K using a liquid cell.^23^ Figure 4 displays the complex impedance plane plot from the representative example **1**:LiTFSI (8:2) in the Col_h_ mesophase at 380 K upon cooling. The presence of a single symmetrical semicircle at intermediate and high frequencies with a resistivity value of 3.21 × 10^5^ Ω·cm reveals a charge transport basically homogeneous in nature, which may indicate the formation of continuous nanochannels during the isotropic liquid–mesophase phase transition. The data in the frequency range of the single semicircle could be well-fitted by using an equivalent circuit model consisting of one standard parallel resistor (R)–constant phase element (CPE) circuit (R1-CPE1), where the CPE accounts for the slight suppression of the semicircle center below the real *Z*′ *x*-axis.

**Figure 4:**
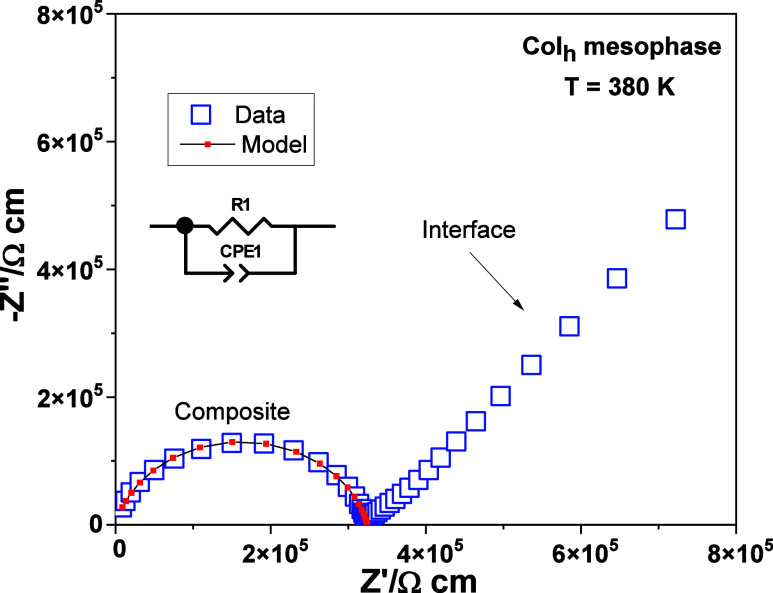
–*Z*″ vs *Z*′ plot for **1**:LiTFSI (8:2) in the hexagonal columnar mesophase at 380 K. The equivalent circuit model based on one R-CPE element is shown.

This, in turn, is commonly associated with non-ideal dielectric response of the sample due to a broadening of the distribution of relaxation times. Besides, the characteristic pike-like dielectric contribution associated with the charge blocking sample–electrode interface contribution can be clearly observed at medium and low frequencies, consistent with predominantly lithium-ion conduction.^33^

The effect of the solid–mesophase phase transition on the charge transport and dielectric properties can be better detected upon heating because the supramolecular ordering changes more notably than on cooling. As demonstrated in Figure 5a, the resistivity in sample **1**:LiTFSI (8:2) decreases by increasing the temperature to the melting point, where the solid–Col_h_ mesophase transition occurs. Across the transition, the resistivity increases, most likely as a consequence of the reorganization of the supramolecular structure. Once the Col_h_ mesophase is fully formed, continuous nanochannels are opened and resistivity again decreases with the increase of temperature as expected in a thermally activated Li-ion charge transport.

**Figure 5:**
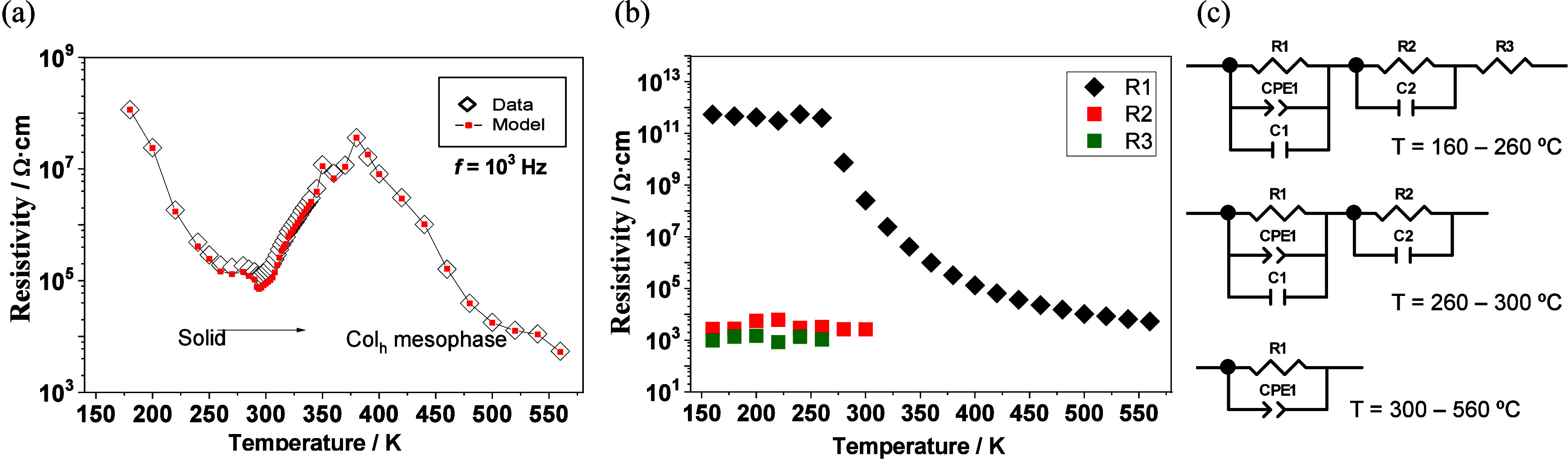
Resistivity as a function of temperature for **1**:LiTFSI (8:2) obtained from (a) the real part of the impedance *Z*′ on heating at a frequency *f* of 10^3^ Hz, where *Z*′ is independent of *f*, and (b) resistivity obtained from equivalent circuit fitting of the data on cooling, showing the three resistors separately. (c) Equivalent circuit models used to fit the data on cooling.

In contrast, upon cooling, the resistivity gradually increases up to the formation of the solid phase at *ca*. 25 °C, which maintains a more similar supramolecular organization like in the mesophase (Figure 5b).^22^ Between 260 and 300 °C, semicircles in the *Z*″ vs *Z*′ plots are more strongly suppressed and slightly asymmetric, which is accounted for by an R-CPE-C element and an additional ideal RC element in series (R2-C2) (Figure 5c). In the solid state between 160–260 °C, an additional dielectric contribution appears, which is accounted for by an additional resistor (R3) with a resistivity of ∼10^3^ Ω·cm that may be associated with heterogeneous conduction of Li^+^ ions through disordered nanochannels.

The real parts of the dielectric permittivity ε′ and the ac conductivity σ′ for the composite **1**:LiTFSI (8:2) are plotted in Figure 6a,b as a function of frequency (*f*) and temperature (*T*) on cooling.

**Figure 6:**
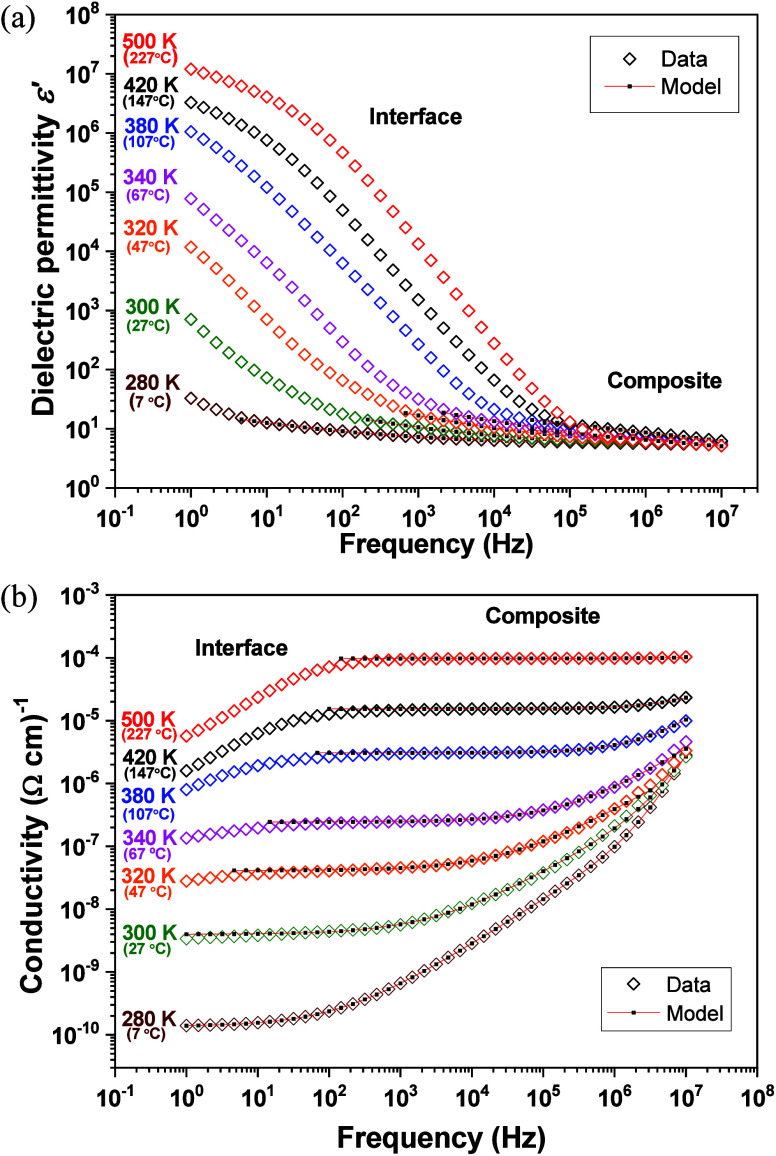
Real parts of the dielectric permittivity ε′ and conductivity σ′ from the **1**:LiTFSI (8:2) sample: (a) ε′ vs *f* curves at various fixed *T*. (b) σ′ vs *f* curves at various fixed *T*.

No drastic changes in the dielectric permittivity are indicated at intermediate and high frequencies across the solid–Col_h_ mesophase transition. Note that the ionic nature of the charge carriers is again supported by the sharp increase in ε′ at low and intermediate frequencies. The high resistivity as a result of the charge blocking at the electrode–sample interface is manifested at high temperatures in the conductivity vs frequency curves in terms of a perceptible drop in σ′ at low frequencies. The Li^+^ conduction through the nanochannels formed in the mesophase reaches maximum values in the order of 10^–4^ Ω^–1^ cm^–1^.

A comparative analysis of the Li-ion conductivity in the Col_h_ mesophase is presented in Figure 7 for all doped **1**:LiTFSI compounds. The doping effect is clear, where the incorporation of lithium ions significantly increases the ionic conductivity by 4 orders of magnitude, reaching a maximum value of 1.89 × 10^–4^ Ω^–1^ cm^–1^ at 560 K for the composite with a 8:2 (**1**:LiTFSI) molar ratio. The activation energies *E*_A_ were obtained from the Arrhenius plots of ln­(σ) vs 1/*T* on cooling (Table S1). In comparison with the *E*_A_ of **1** (0.83 eV), the presence of Li-ions causes a clear decrease in the *E*_A_ values with the unique exception of the composite with a 8:8 (**1**:LiTFSI) molar ratio, which has a higher value of 0.90 eV. Interestingly, results seem to indicate that there exists a direct correlation between the Li content and the activation energies, as also observed for conductivity values. The lowest *E*_A_ with a value of 0.41 eV is found precisely for the composite **1**:LiTFSI (8:2) that simultaneously reaches the highest ionic conductivity (Figure 7b). To understand the origin of these results, it is necessary to first investigate the lithium conduction mechanism.

**Figure 7:**
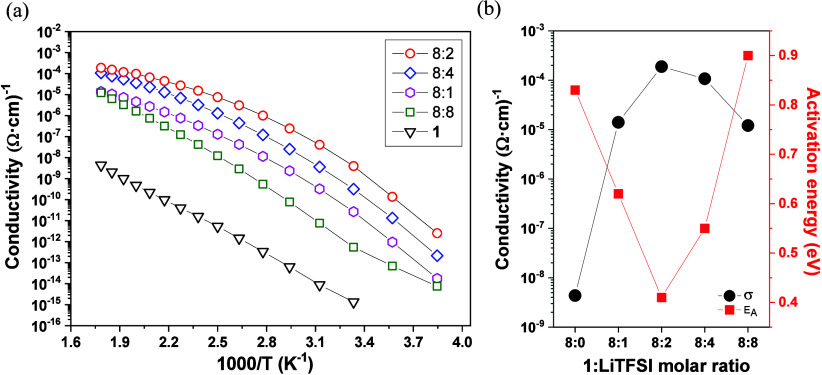
(a) Conductivity vs 1000/*T* curves in the temperature range of the Col_h_ mesophase for all composites fabricated with the **1**:LiTFSI molar ratio, as indicated. Compound **1** corresponds to the undoped variant. The conductivity values were obtained from the equivalent circuit fits. (b) Maximum conductivity reached in the mesophase at 560 K and activation energies values, *E*_A_, as a function of the **1**:LiTFSI molar ratio.

As mentioned above, XRD and POM studies revealed that the presence of lithium ions induces the formation of a more stable and ordered Col_h_ mesophase, most likely due to the establishment of ion–dipole interactions between the lithium ions and pyrazolate nitrogen atoms of the Pd­(II) compounds. From a slightly speculative point of view, it may be argued that the proton conduction mechanism detected in the undoped compound **1** reported previously may be hindered by the Li-doping,^22^ since the above-mentioned ion-dipole interactions would hamper the proton transfer between the pyrazolate nitrogen atom of a ligand and the nearest isoquinolinyl proton of the other coordinated ligand (see Figure 8a). On the other hand, lithium ions are likely to be mobile and transfer easily via the continuous nanochannels of the ordered Col_h_ mesophase. The activation energies calculated are consistent with this hypothesis. The proton conduction in **1** was found to be associated with a C–H···N proton transfer that requires high activation energies of 0.83 eV, whereas the *E*_A_ in the new composites decreases to values of 0.41 eV, most likely due to the suppression of that proton transfer. To shed light on this hypothesis, density functional theory (DFT) calculations were performed to analyze the structural changes induced in **1** after Li-ion doping.

**Figure 8:**
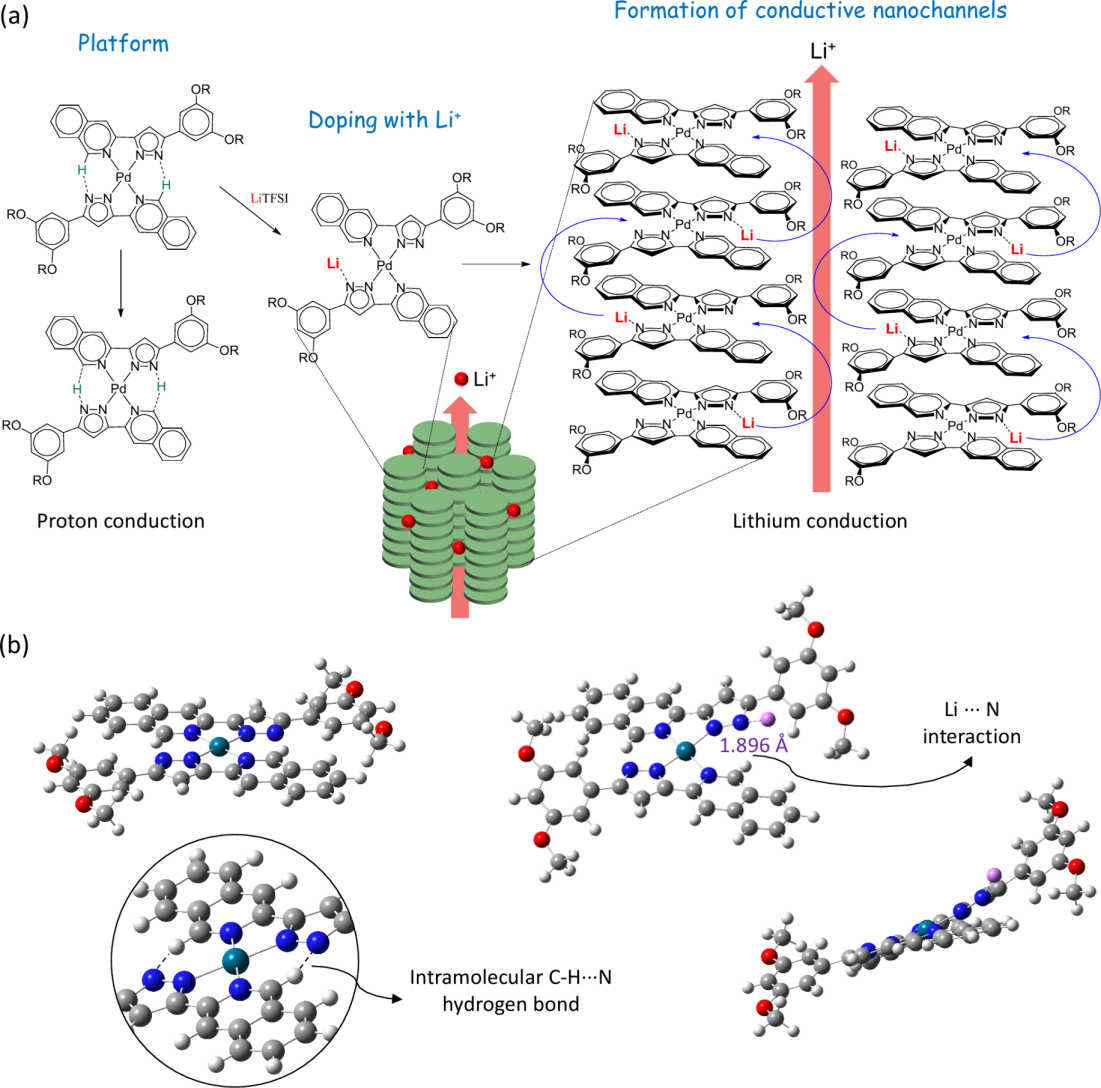
(a) Proposed lithium ion conduction mechanism for **1**:LiTFSI composites in the Col_h_ mesophase. (b) DFT optimized structures of the Pd­(II) compound and the lithium-ion doped Pd­(II) species (Pd, green; N, blue; O, red; C, gray; Li, pink; H, white).

Figure 8b displays the optimized structure of **1** (−62597.78 eV). As expected, the overall molecule shows a high planarity because of the square-planar coordination environment around the palladium center and the establishment of interligand C–H···N hydrogen bonds between the two coordinated pyrazolate ligands (d­(N···H): 2.105 Å, <(C–H···N): 148.5°). However, calculations considering that Li^+^ is interacting with the pyrazolate nitrogen atom reveal a loss of planarity of the molecule and, concomitantly, the rupture of those C–H···N hydrogen bonds (−62792.66 eV). These results are consistent with the proposed lithium conduction mechanism and confirm that the proton transfer may be suppressed in the composites.

Apparently, the lithium content plays a crucial role in the Li-ion conductivity. As demonstrated in Figure 7b, a low lithium molar ratio gives rise to low conductivity values as would be expected for a small concentration of Li^+^ charge carriers. Similarly, low ionic conductivity is found when the composite is Li-doped to a high molar ratio of 8:8 (**1**:LiTFSI). This implies that the Li^+^ ions are less mobile and seem to have difficulty moving freely along the nanochannels. In fact, note that the activation energy reaches the highest value of 0.90 eV for this composite. This may be explained by the fact that a high number of Li^+^ charge carriers would leave few vacant Li^+^-positions, which may be located near the pyrazolate nitrogen atoms (see Figure 8). However, such Li^+^ acceptor positions or Li^+^ vacancies are required for the lithium ions to jump into when they transfer from a neighboring molecule along the nanochannels. Since there are two free pyrazolate nitrogen atoms that can act as acceptor states or Li^+^ vacancies in each molecule, the maximum doping level applied (8:8/**1**:LiTFSI) corresponds to the same number of Li^+^ vacancies and Li^+^ ions. However, this seems to be unfavorable for Li-ion transport, and the highest conductivity is achieved for composites with intermediate 8:2 and 8:4 (**1**:LiTFSI) molar ratios, with ionic conductivity values of up to 1.89 × 10^–4^ Ω^–1^ cm^–1^ and activation energies of 0.41 eV. This indicates that Li^+^ ions may exhibit higher mobility than Li^+^ vacancies, as expected.

Furthermore, it may be argued that the increasing Li^+^ content in the 8:4 and 8:8 composites may lead to a high concentration of TFSI^–^ anions, which, for steric reasons, may most likely be situated near or within the nanochannels. This argument is supported by the fact that the intercolumnar distance between the disc-like molecules of neighboring columns in terms of the lattice constant is hardly affected by the Li^+^ doping level as mentioned above (Table 1). In addition, the increase of the long-range order in the Col_h_ mesophase with LiTFSI doping would hinder the cooperative motions of disc-like molecules,^23^ which constitute a drawback for the lithium ions to jump between the Li^+^ vacancies of neighboring molecules. Thus, it is likely that all of these factors contribute to the observed reduction in Li^+^ ion conductivity for the 8:8 (**1**:LiTFSI) composite.

## Conclusions

A bis­(isoquinolinylpyrazolate)­Pd­(II) metallomesogen (compound **1**) has been used as a platform to prepare Li-doped hexagonal columnar nanoassemblies by the incorporation of the LiTFSI salt. The novel composites form liquid crystalline phases at room temperature that are stable up to 400 °C. The formation of ion–dipole interactions between the Li^+^ ions and the noncoordinated pyrazolate nitrogen atoms of the ligands are proposed to be responsible for the stabilization of the columnar mesophases over wider temperature ranges. These interactions restrain the axial fluctuations of molecules and increase the long-range order in the mesophase, creating a network of one-dimensional nanochannels in the supramolecular assemblies that appear to be suitable for high lithium-ion conduction. The incorporation of lithium ions significantly enhances the ionic conductivity, reaching values of up to 1.89 × 10^–4^ Ω^–1^ cm^–1^ for the composite **1**:LiTFSI (8:2), whereas higher 8:4 and 8:8 (**1**:LiTFSI) molar ratios show lower conductivity. This may be related to a lower concentration of vacant Li^+^ sites near the pyrazolate nitrogen atoms, and/or the increased amount of TFSI^–^ anions blocking the nanochannels for Li^+^ ion transport.

The work presented demonstrates that metallomesogens can serve as a platform for the preparation of liquid crystalline composites that act as Li-ion conductors, albeit currently at high operational temperatures. Interestingly, the composites investigated may constitute a new class of materials for potential applications as electrolytes. Furthermore, the results described herein pave the way for the design of new metallomesogens with other metal centers, such as Zn­(II) and Cu­(II), aligning mesophases to improve the Li-ion conductivity, that may also be extended to other interesting ions such as Na^+^, K^+^, or Mg^2+^. It is expected that these findings will further stimulate the development of new metallomesogenic materials with high ionic conductivity at lower temperatures, enabling their use in metal-ion batteries.

## Experimental Section

### Starting Materials

Solvents were purchased from Scharlab and used without further purification. LiTFSI was purchased from Merck. The Pd­(II) metallomesogen, compound **1**, was synthesized as previously reported.^22^

### Synthesis of Li-Doped Liquid Crystals

Several THF solutions containing Pd­(II) metallomesogen compound **1** and LiTFSI in the desired molar ratio were prepared. THF was evaporated at room temperature for 24–48 h, yielding a sticky yellow solid that was further dried at 80 °C for 24 h before use.

### Physical Measurements

The mesomorphic behavior of the Li-doped liquid crystal composites was analyzed by POM and XRD studies. POM observations were carried out using an Olympus BX50 microscope equipped with a Linkam THMS 600 heating stage and a digital camera DP28. Small-angle powder XRD studies were carried out at variable temperature on a Panalytical X’Pert PRO MPD diffractometer with Cu–Kα (1.5406 Å) radiation in a θ–θ configuration equipped with an Anton Paar HTK1200 heating stage, from the X-ray Diffraction Service of the Complutense University of Madrid.

^7^Li MAS NMR (magic angle spinning nuclear magnetic resonance) spectra were recorded at room temperature in an AVANCE III HD 600 (Bruker AXS) spectrometer by using a triple resonance DVT probe of 2.5 mm at a spinning rate of 20 kHz. The magnetic field was 14.1 T, corresponding to a ^7^Li resonance frequency of 233.21 MHz. The ^7^Li chemical shifts are referenced to LiCl at 5 M. ^7^Li MAS NMR spectra were recorded with a 0.25 μs 90° pulse and a 3 s delay and summing up 500 scans.

X-ray photoelectron spectroscopy (XPS) measurements were carried out on a PHI Versa-Probe II Scanning XPS Microprobe (Physical Electronics) spectrometer. Data collection was obtained using a scanning monochromatic Al Kα X-ray source (*h*ν = 1486.6 eV) and a charge neutralizer operating at 100 W and 20 kV under a vacuum of 10^–7^ Pa. The XPS spectra were fitted by using a PHI SmartSoft software and processed using the MultiPak 9.3 package. The C 1s at 284.8 eV from the adventitious carbon layer was used to calibrate the spectra and the binding energy shifts of the solids.

Thermal analyses were carried out with a PerkinElmer Pyris 1 differential scanning calorimeter. Samples were hermetically sealed in aluminum pans, and measurements were carried out in a N_2_ atmosphere with heating and cooling rates of 10 K min^–1^. TGA experiments were obtained on a PerkinElmer Pyris 1 thermogravimetric analyzer under the same conditions (heating rate of 10 K min^–1^ and N_2_ atmosphere).

DFT calculations were performed with the Gaussian 9.0 software package.^34^ The geometry optimization was performed at the B3LYP level, employing the SDD (Stuttgart/Dresden Effective core potentials) basis set with an effective core potential (ECP) to describe the transition metal and the 6-31G­(d) basis set for all other atoms.^35,36^ Alkyl chains were replaced by methyl groups to reduce the computational time. The results were visualized using Gauss-View 5.0 software.

The ionic charge transport and dielectric properties of the compounds in the solid phase and the Col_h_ mesophase were studied by alternating current (AC) impedance spectroscopy using an Alpha Analyzer integrated into a Novocontrol BDS 80. Measurements were performed at a frequency (*f*) range of 1 Hz to 10 MHz with six measurements points per frequency decade, using a 0.1 V amplitude for the applied AC voltage signal. The temperature (*T*) was varied between 160 K and the upper instrumental limit of 560 K (−113 to 287 °C) upon heating and cooling cycles. Dielectric data were taken under steady state conditions, i.e., the temperature was stabilized for 3–10 min before taking impedance measurements over the full *f*-range. The temperature increments/reductions for taking impedance measurements were 20–2 K steps. In particular, the temperature was increased/decreased in smaller steps near the solid-mesophase phase transition. The composites in the solid state were placed between the polished electrodes of a custom-built stainless-steel liquid cell with a high surface to thickness ratio.^23^ The cell was closed with a sapphire plate and placed inside of the Novocontrol cryostat.

The dielectric response of the materials was obtained at the selected temperatures for heating and cooling cycles in terms of the real and imaginary parts (*Z*′, *Z*″) of the complex impedance Z* = *Z*′ + *iZ*″. The data were converted into the complex conductivity σ* and capacitance ε* notations, σ* = σ′ + *i*σ″ and ε* = ε′ – *i*ε″, using the standard conversions: Z* = (*g*σ*)^−1^, and Z* = (*i*ωε*)^−1^, where *g* (in cm) is the geometrical factor given by electrode area divided by electrode distance and ω is the angular frequency. The geometrical factor *g* could only be estimated from the weight and density of the powder measured initially and the dimensions of the liquid cell. Equivalent circuit fitting of the dielectric data was performed by using Z-View software and a custom-built automated data analysis tool based on two advanced Microsoft Excel macros.

## Supplementary Material



## Notes

XRD diffractogram in the mesophase; differential scanning calorimetric thermograms; thermogravimetric analysis; and activation energy *E*_A_ values (PDF)
