# Supplementary material for: Lithium-Ion Conduction in Liquid-Crystalline Columnar Pd(II) Nanoassemblies
Source: ACS Appl Mater Interfaces. 2025 Jun 12;17(30):42915–24. doi: 10.1021/acsami.5c00209 (PMC12314862; doi:10.1021/acsami.5c00209)
Supplement: Supplementary file 1 [file am5c00209_si_001.pdf]

Supporting Information

Lithium-ion conduction in liquid-crystalline columnar Pd(II) nanoassemblies

*Cristián Cuerva[a, *], Irene Caro-Campos[a], Mercedes Cano[a], Enrique Rodríguez-Castellón[b], Alois Kuhn[c], Flaviano García-Alvarado[c], Rainer Schmidt[d, *]*

[a] *Departamento de Química Inorgánica, Facultad de Ciencias Químicas, Universidad Complutense de Madrid, Ciudad Universitaria, E-28040 Madrid, Spain.*

[b] *Departamento de Química Inorgánica, Facultad de Ciencias, Instituto Interuniversitario de Investigación en Biorrefinerías I3B, Universidad de Málaga, 29071 Málaga, Spain.*

[c] *Departamento de Química y Bioquímica, Facultad de Farmacia, Universidad San Pablo-CEU, CEU Universities, Urbanización Montepríncipe, Boadilla del Monte, Madrid 28668, Spain.*

[d] *GFMC. Departamento de Física de Materiales, Facultad de Ciencias Físicas, Universidad Complutense de Madrid, Ciudad Universitaria, E-28040 Madrid, Spain.*

*Corresponding autor: Cristián Cuerva. E-mail: c.cuerva@ucm.es

Rainer Schmidt. E-mail: rainer.schmidt@fis.ucm.es

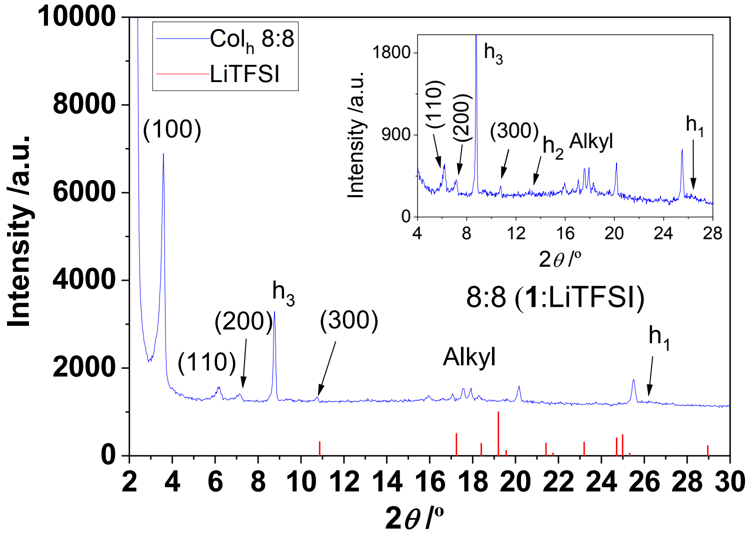

Figure S1. XRD diffractogram in the mesophase of the composite **1**:LiTFSI doped with a 8:8 molar ratio, including reflections of pure LiTFSI (red lines).

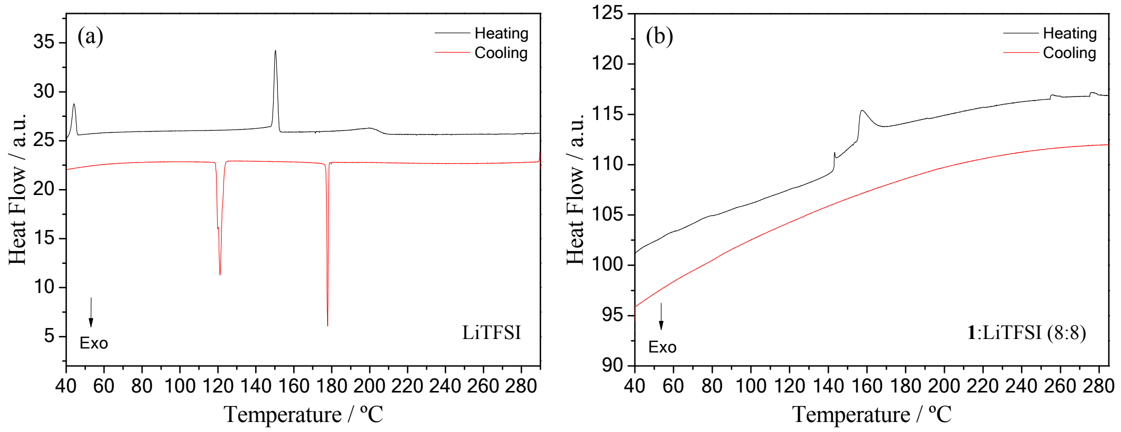

Figure S2. Differential scanning calorimetric thermograms registered for (a) LiTFSI and (b) **1**:LiTFSI (8:8), in the first heating and cooling cycles.

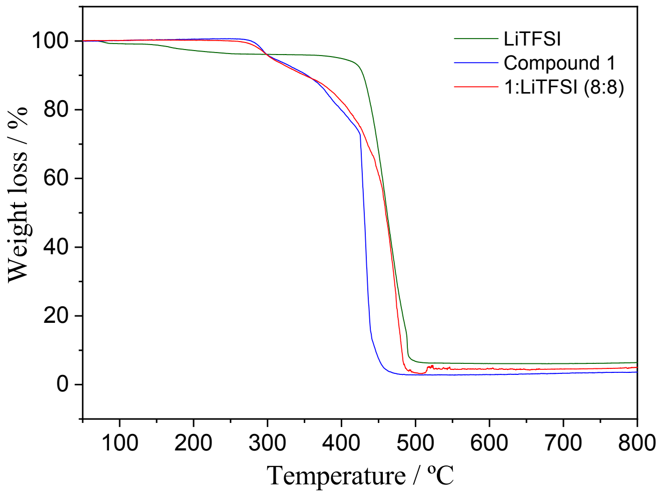

Figure S3. Thermogravimetric analysis for LiTFSI (green), compound **1** (blue) and the composite **1**:LiTFSI (8:8) (red).

Table S1. Activation energies E_A obtained for the composites in the temperature range of the Col_h mesophase.

**1**:LiTFSI molar ratio	Activation energy (eV)
8:0	0.83
8:1	0.62
8:2	0.41
8:4	0.55
8:8	0.90
